# Supplementary material for: Interactions with multiple inner kinetochore proteins determine mitotic localization of FACT
Source: J Cell Biol. 2025 Mar 17;224(5):e202412042. doi: 10.1083/jcb.202412042 (PMC11912937; doi:10.1083/jcb.202412042)
Supplement: Table S1 — summarizes the phospho-sites on SPT16 and SSRP1 that were phosphorylated by CK2 in vitro and could be detected by mass spectrometry. [file jcb_202412042_tables1.docx]

|  | **Position** | **Phospho (STY) Probabilities** | **PEP** | **Score** |
| --- | --- | --- | --- | --- |
| **SPT16**  (untreated) | 455 | NEDEEEEEEEKDEAEDLLGRG**pS**(1)R | 3.44E-61 | 319.77 |
|  | 982 | ES(0.017)LG**pS**(0.983)EEESGKDWDE  LEEEARK | 2.56E-10 | 116.72 |
| **SSRP1** (untreated) | 170 | FY(0.001)VPP**pT**(0.999)QEDGVDPVEA  FAQNVLSK | 1.78E-07 | 102.29 |
|  | 437 | EGMNP**pS**(0.907)Y(0.093)DEYAD  **pS**(1)DEDQHDAYLER | 3.95E-23 | 179.49 |
|  | 444 | EGMNPSYDEYAD**pS**(1)DEDQHDAYLER | 5.92E-140 | 447.46 |
|  | 578 | SDHPGISITDL**pS**(1)K | 4.47E-09 | 127.71 |
|  | 657 | QL**pS**(1)ESFK | 7.26E-04 | 108.74 |
|  | 667 | EFV**pS**(0.998)S(0.001)DESSSGENK | 1.11E-12 | 189.23 |
|  | 672 | EFVSSDES(0.194)**pS**(0.671)  S(0.135)GENK | 3.18E-12 | 178.23 |
| **SPT16**  **CK2** | 979; 986 | E**pS**(0.977)LGS(0.023)EEE  **pS**(1)GKDWDELEEEAR | 4.29E-22 | 170.53 |
|  | 1004 | ADRE**pS**(1)RYEEEEEQSR | 2.96E-13 | 190.55 |
| **SSRP1**  **CK2** | 437 | EGMNP**pS**(0.731)Y(0.093)DEYAD  **pS**(1)DEDQHDAYLER | 2.97E-16 | 160.43 |
|  | 438 | EGMNPS(0.292)**pY**(0.707)DEY(0.002)  AD**pS**(0.999)DEDQHDAYLER | 7.50E-09 | 112.99 |
|  | 444 | EGMNPSYDEYAD**pS**(1)DEDQHDAYLER | 2.65E-106 | 389.53 |
|  | 667 | EFV**pS**(0.999)S(0.001)DESSSGENK | 5.17E-29 | 265.89 |

Phosphorylation sites on FACT expressed in insect cells or after treatment with CK2 as detected by mass spectrometry. Identified sites (shown in bold characters) are followed by the probability, the p-value of the posterior error probability (PEP) of the peptide and the Andromeda search engine score. Residues with a score <100 and residues that were also detected in the dephosphorylated sample were excluded.
