# Supplementary figures and images for: Interactions with multiple inner kinetochore proteins determine mitotic localization of FACT
Source: J Cell Biol. 2025 Mar 17;224(5):e202412042. doi: 10.1083/jcb.202412042 (PMC11912937; doi:10.1083/jcb.202412042)

**D**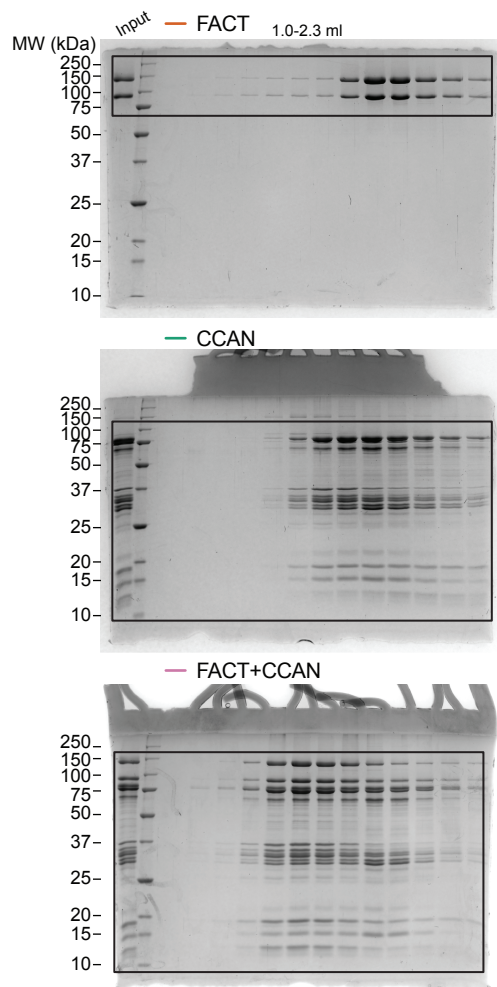**E**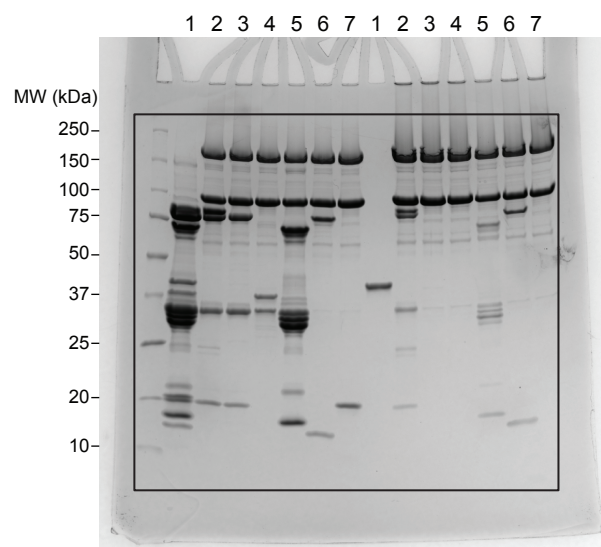

Supplement: SourceData F1 — is the source file for Fig. 1. [file jcb_202412042_sourcedataf1.pdf]

**A**

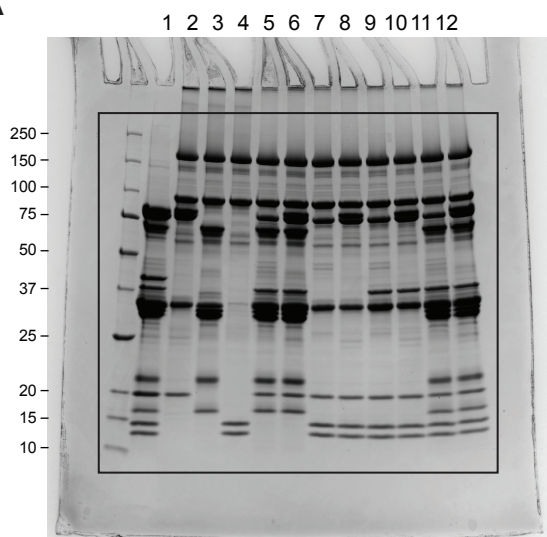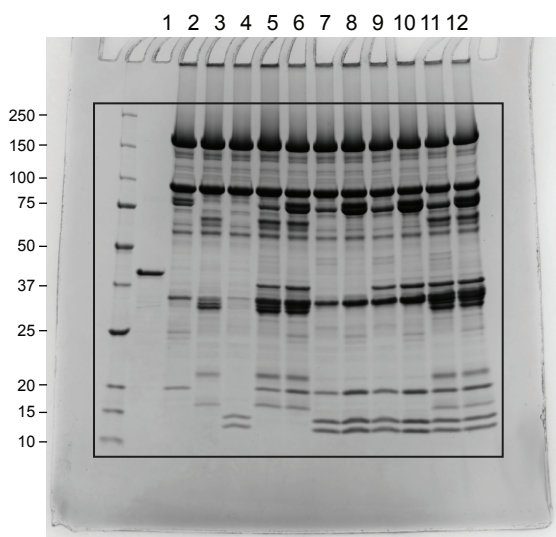

Source Data 3

Supplement: SourceData F3 — is the source file for Fig. 3. [file jcb_202412042_sourcedataf3.pdf]

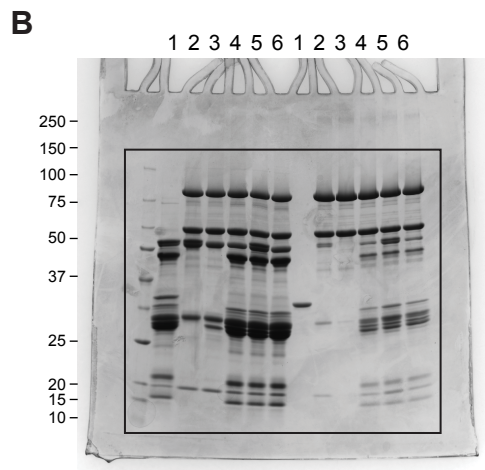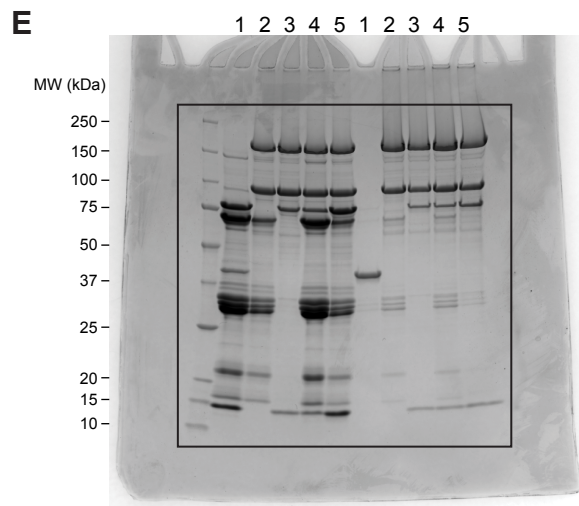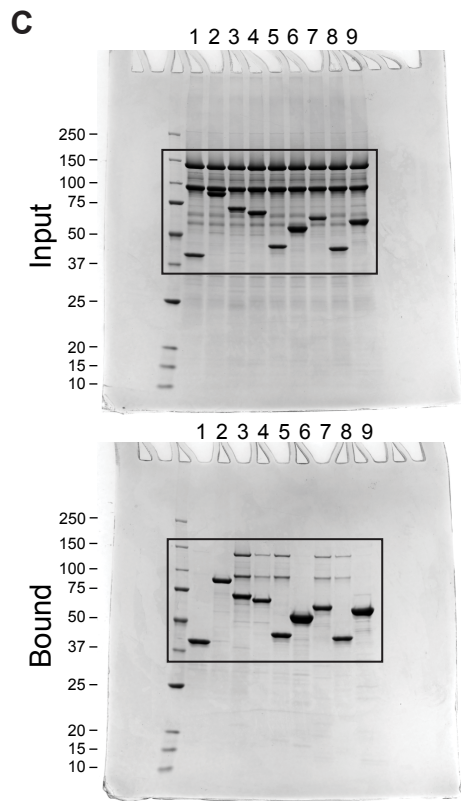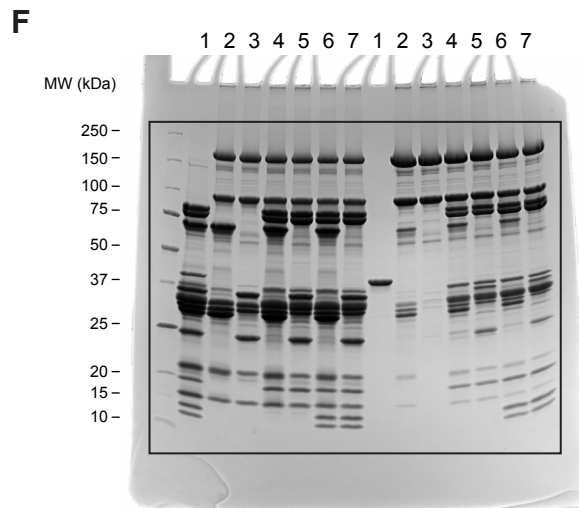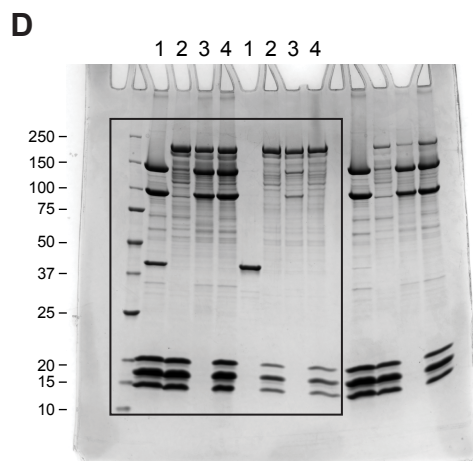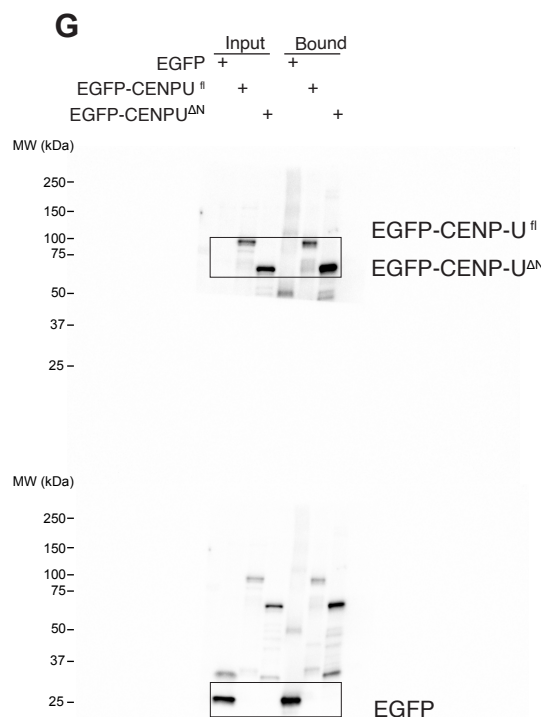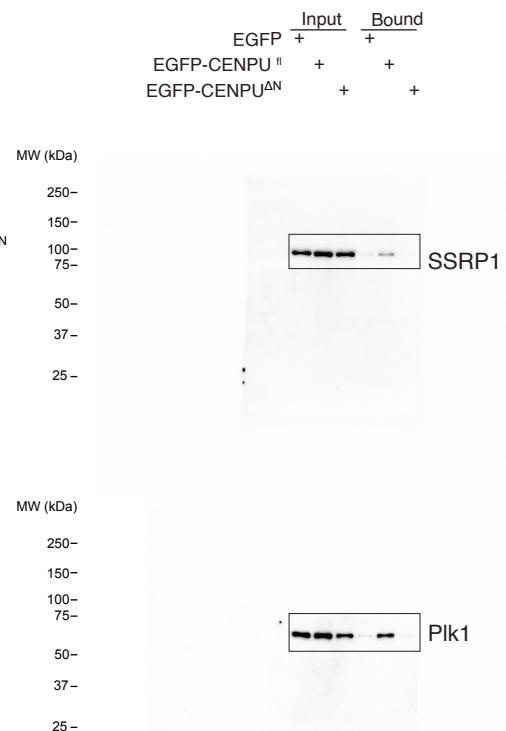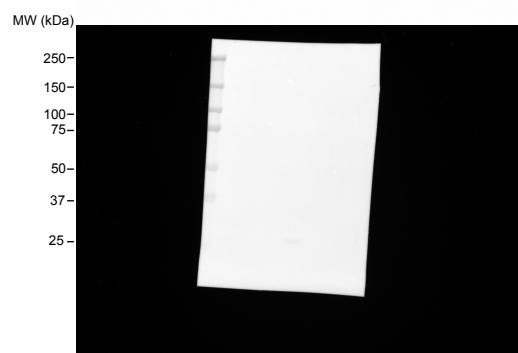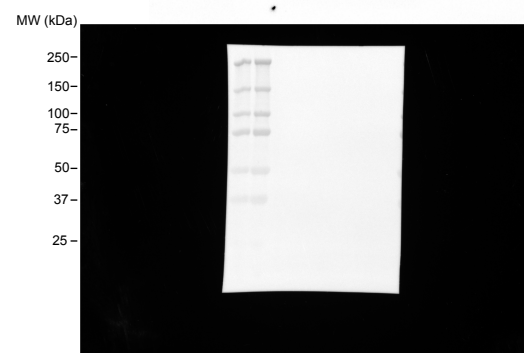

Supplement: SourceData F4 — is the source file for Fig. 4. [file jcb_202412042_sourcedataf4.pdf]

\* unrelated samples

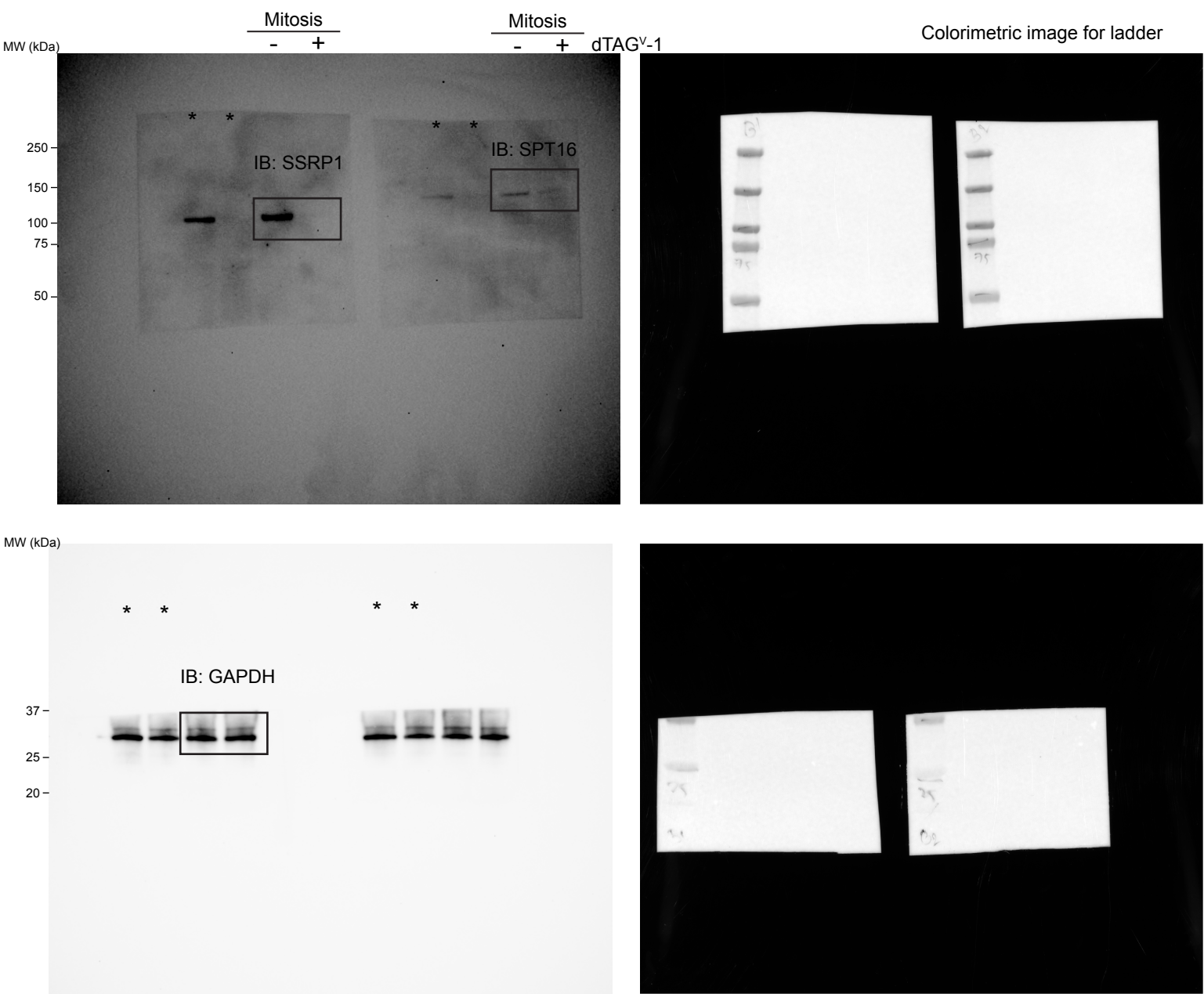

Supplement: SourceData F6 — is the source file for Fig. 6. [file jcb_202412042_sourcedataf6.pdf]

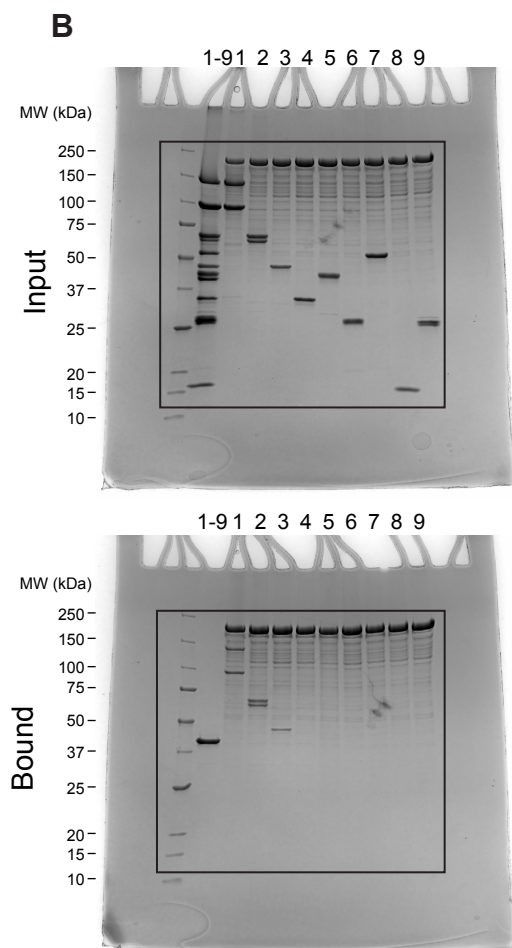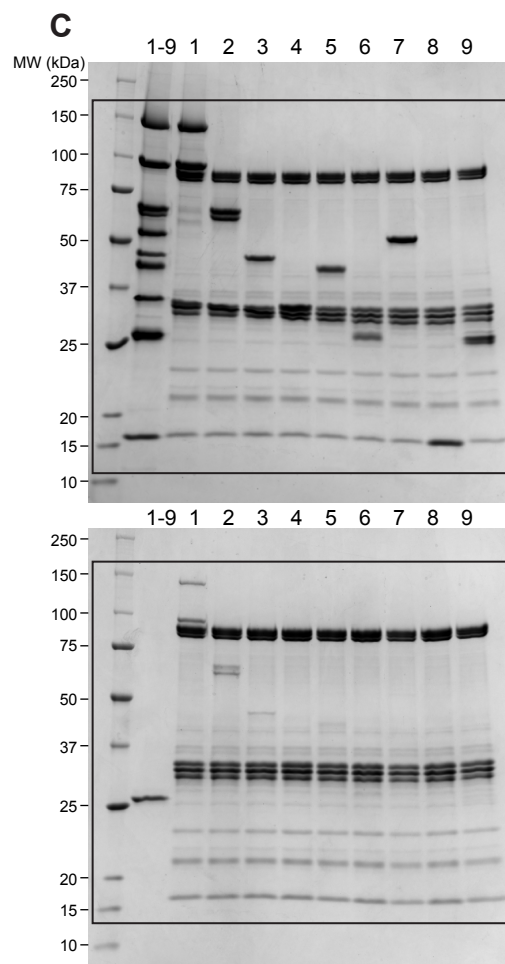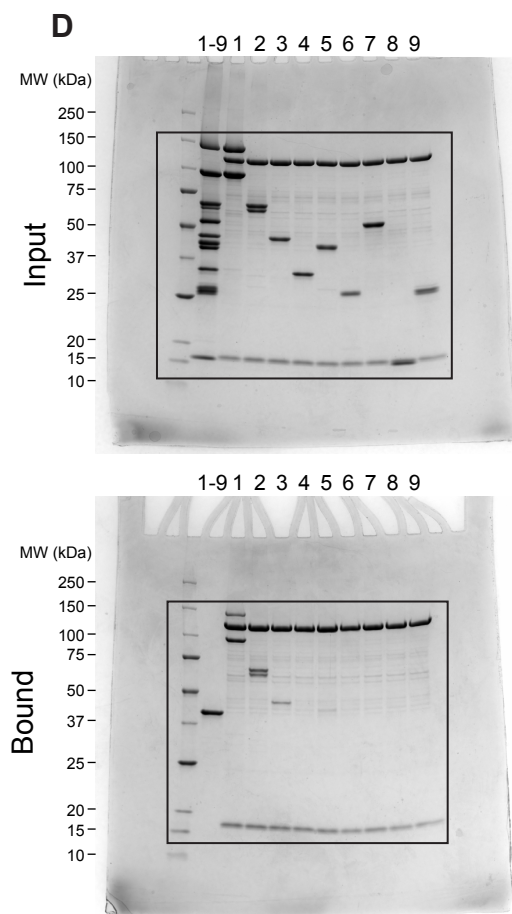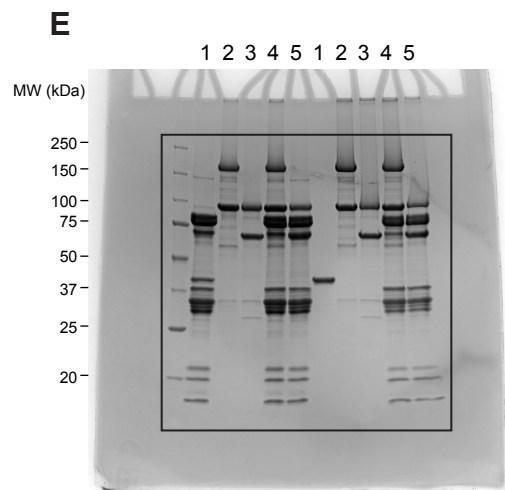

Supplement: SourceData F7 — is the source file for Fig. 7. [file jcb_202412042_sourcedataf7.pdf]

A

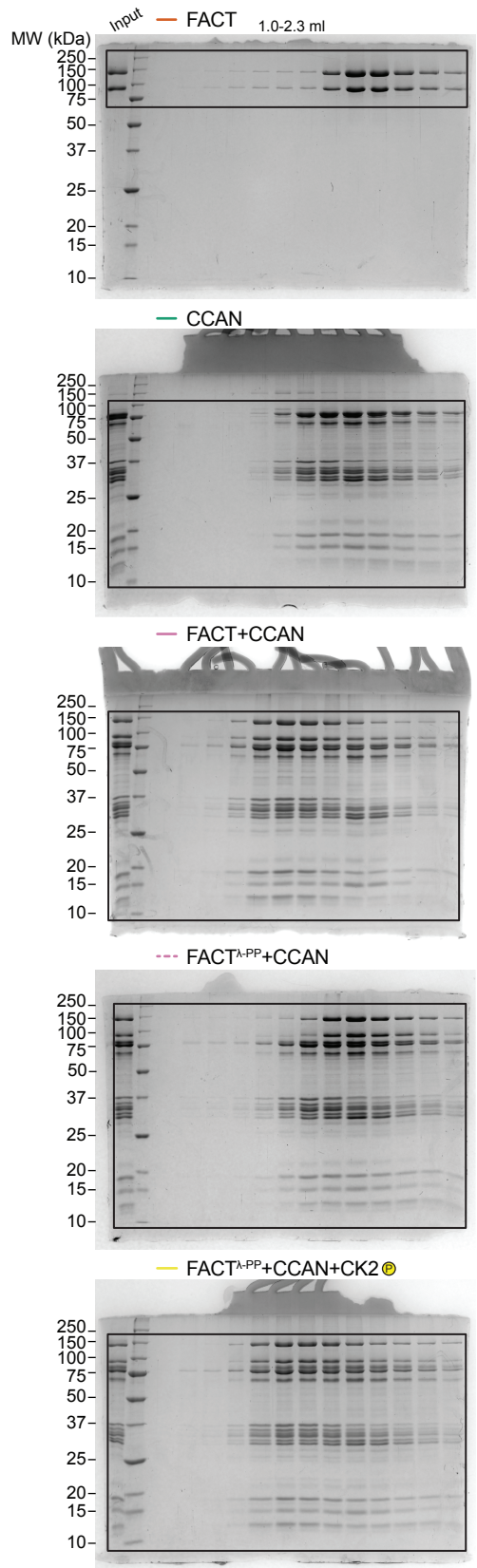

B

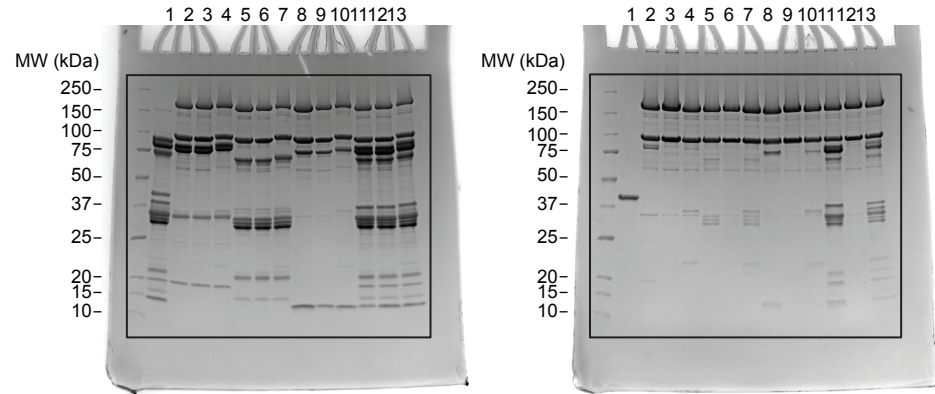

C

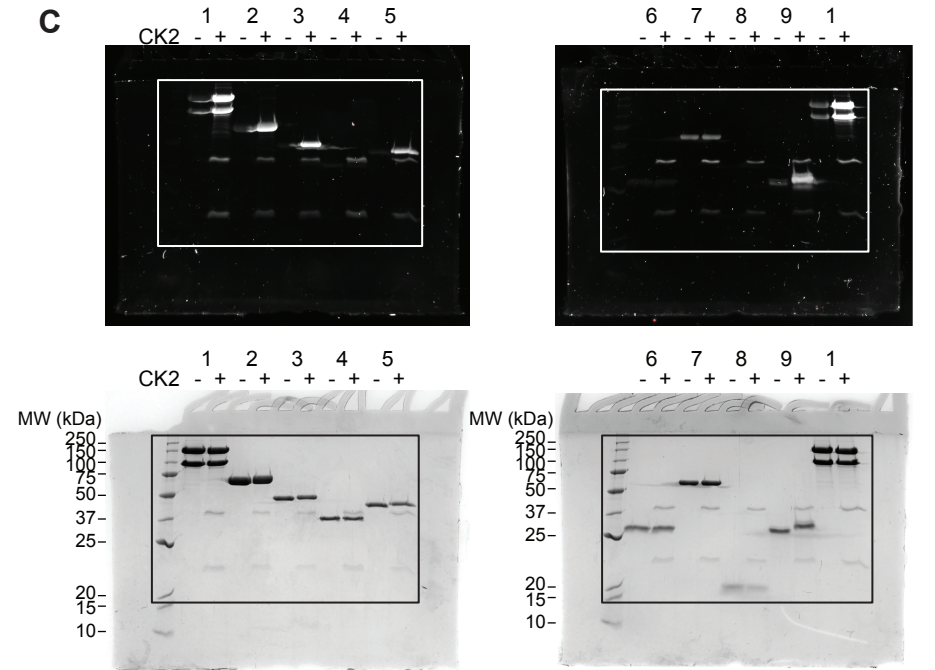

Source Data 8

Supplement: SourceData F8 — is the source file for Fig. 8. [file jcb_202412042_sourcedataf8.pdf]

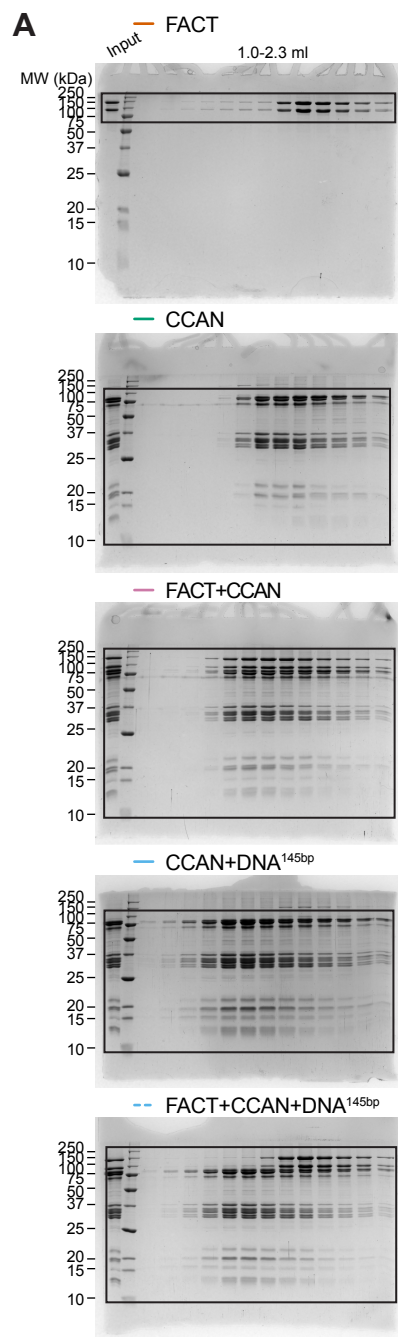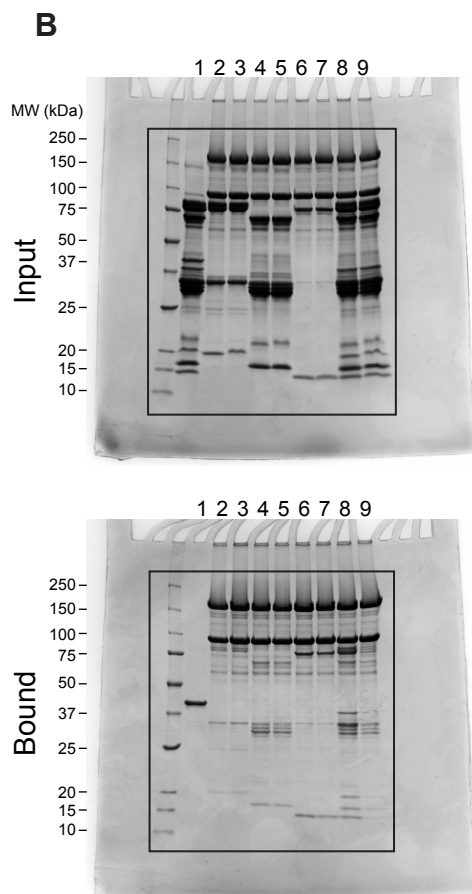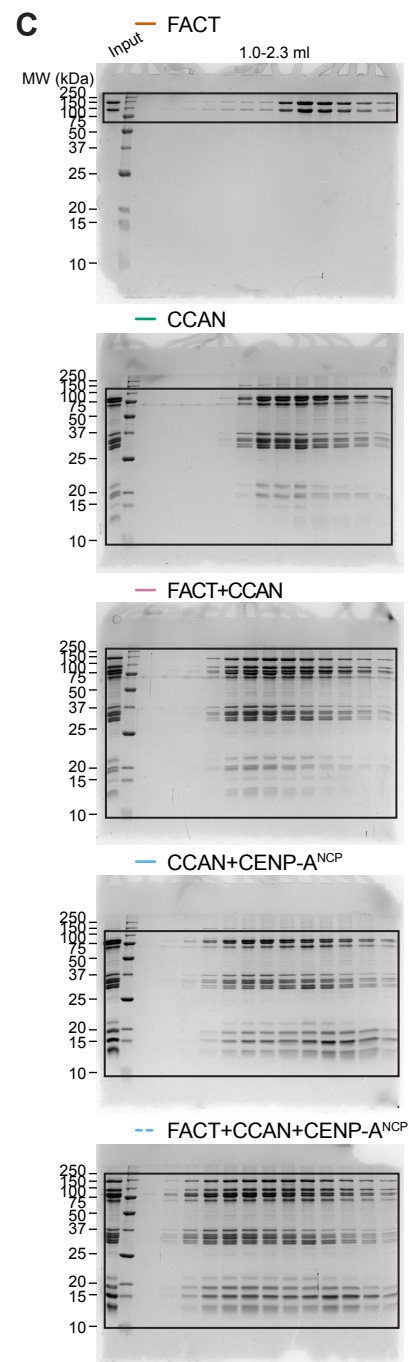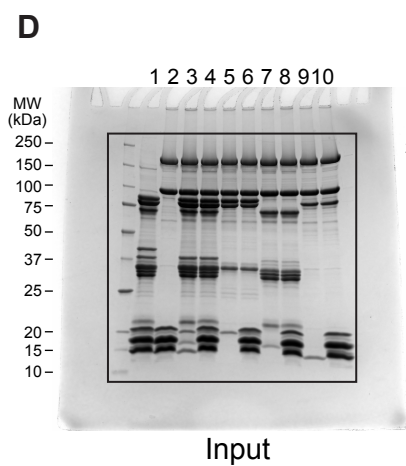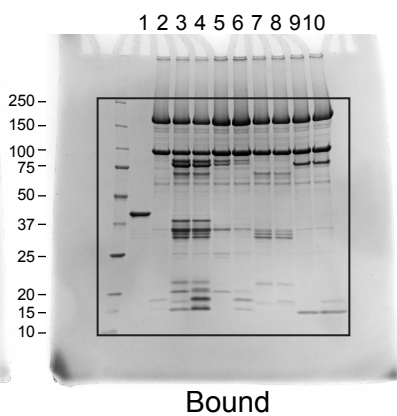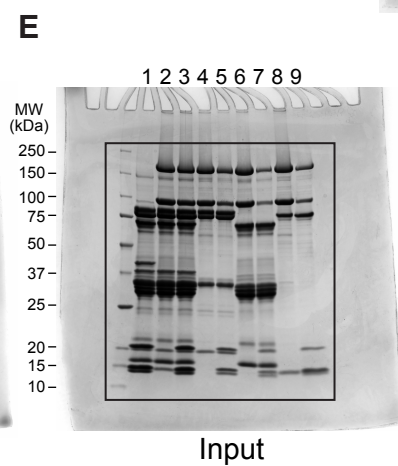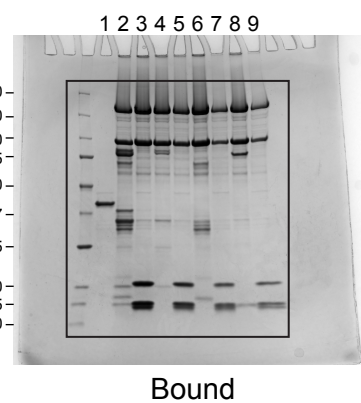

Source Data 9

Supplement: SourceData F9 — is the source file for Fig. 9. [file jcb_202412042_sourcedataf9.pdf]

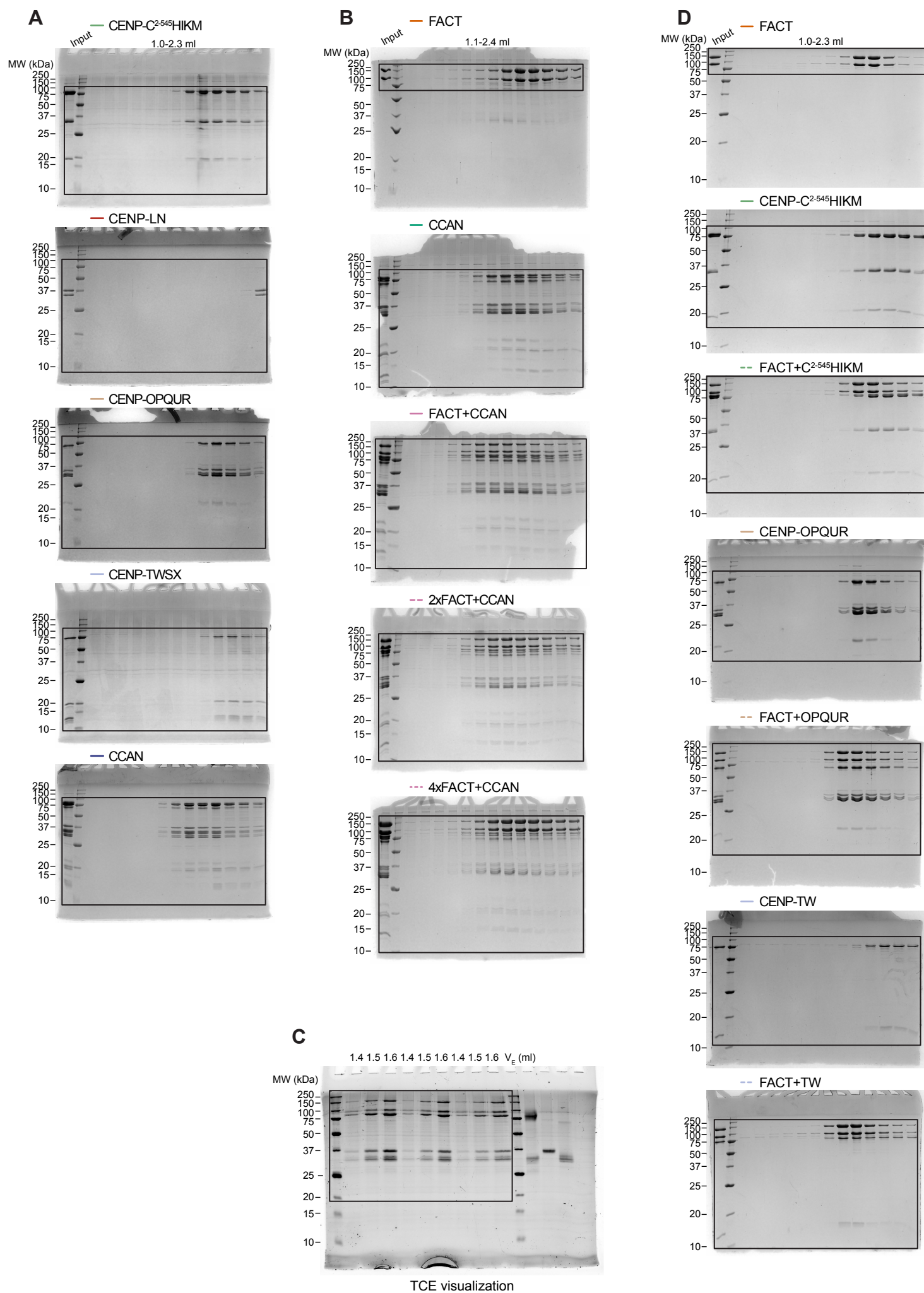

Supplement: SourceData FS1 — is the source file for Fig. S1. [file jcb_202412042_sourcedatafs1.pdf]

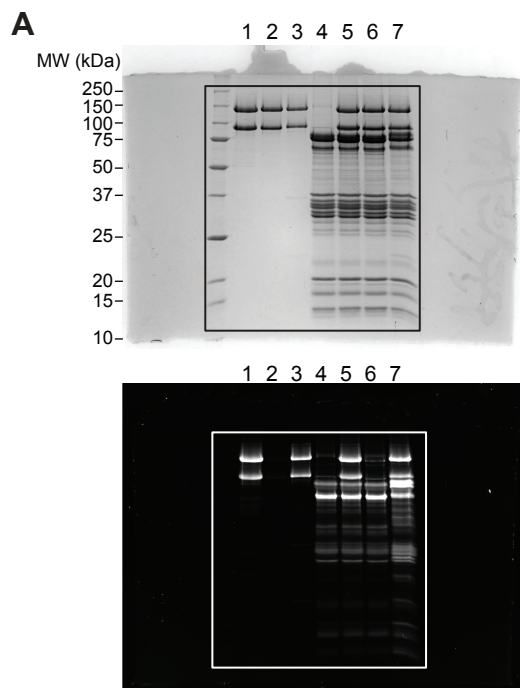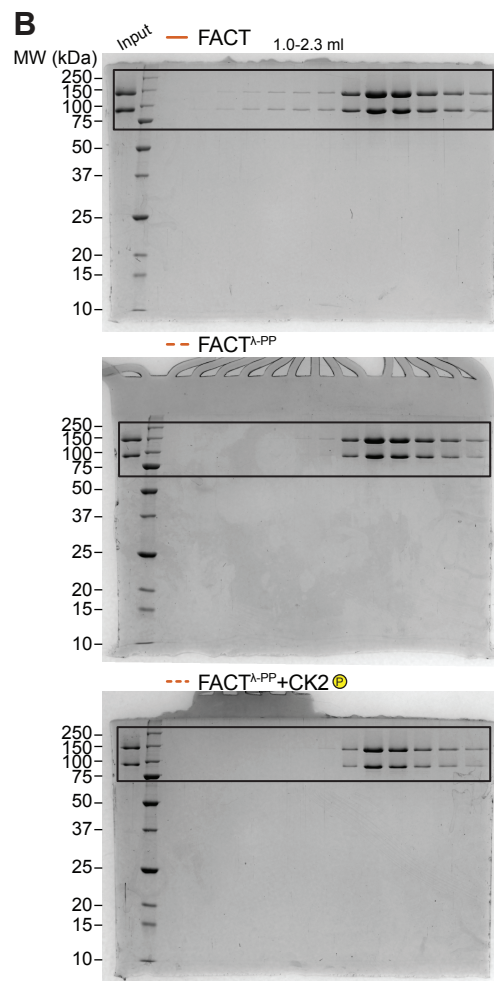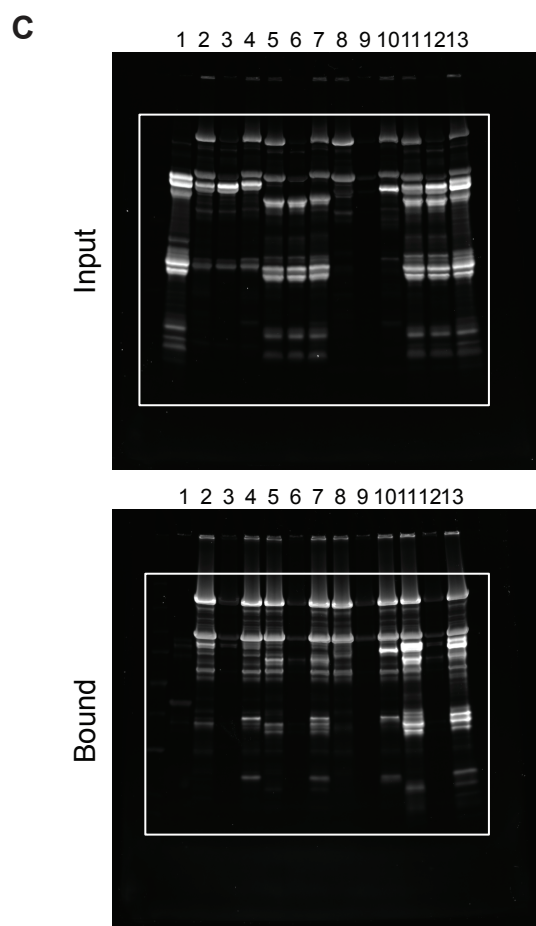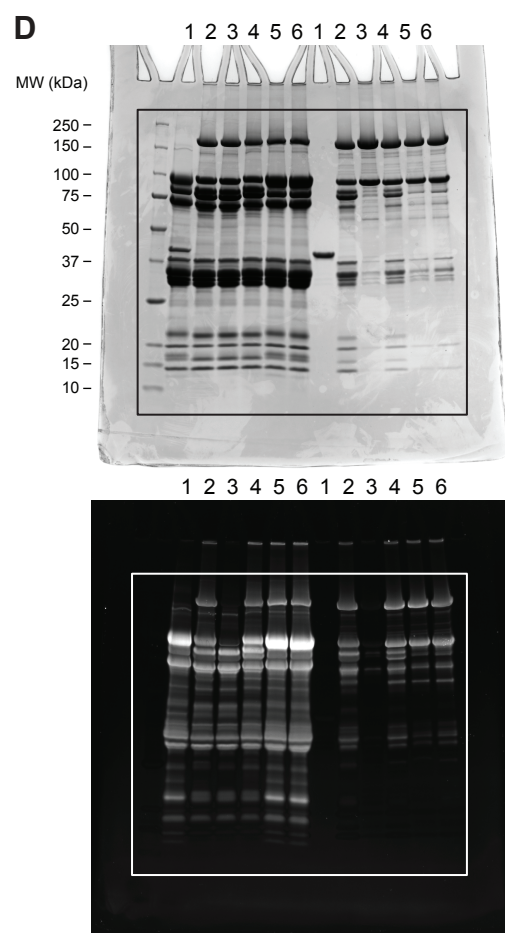

Supplement: SourceData FS5 — is the source file for Fig. S5. [file jcb_202412042_sourcedatafs5.pdf]

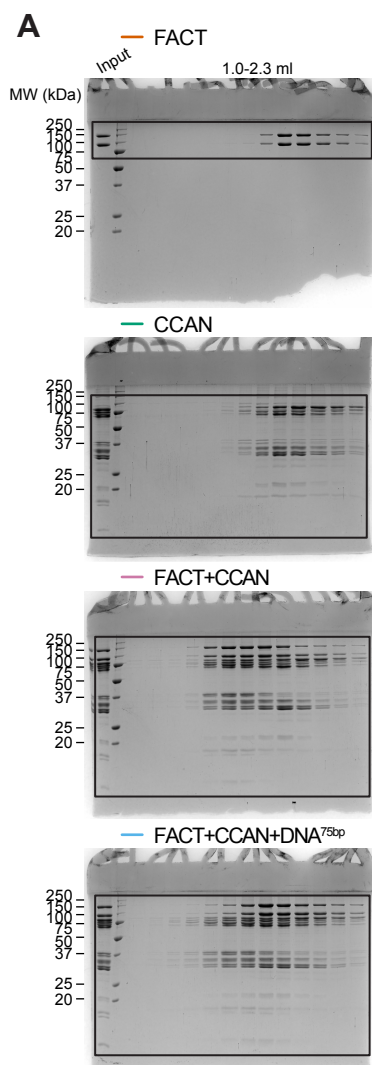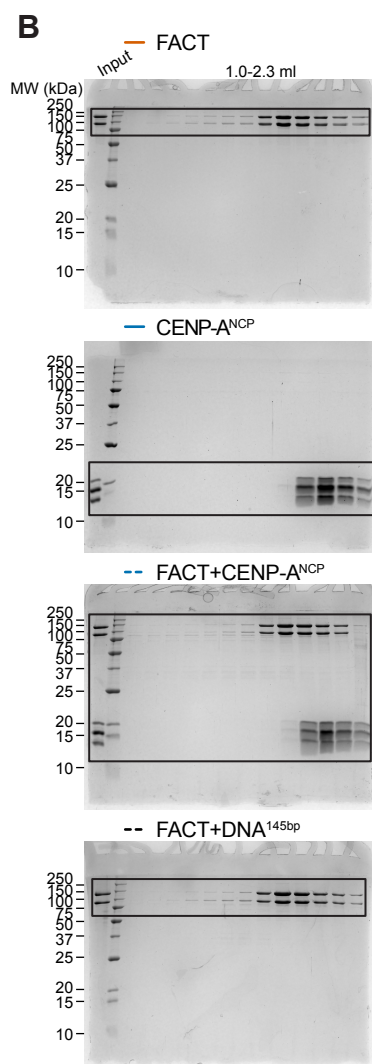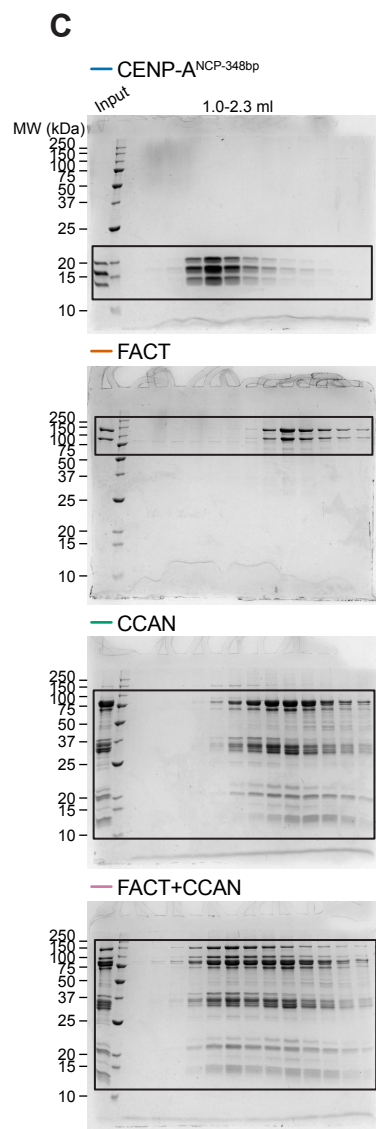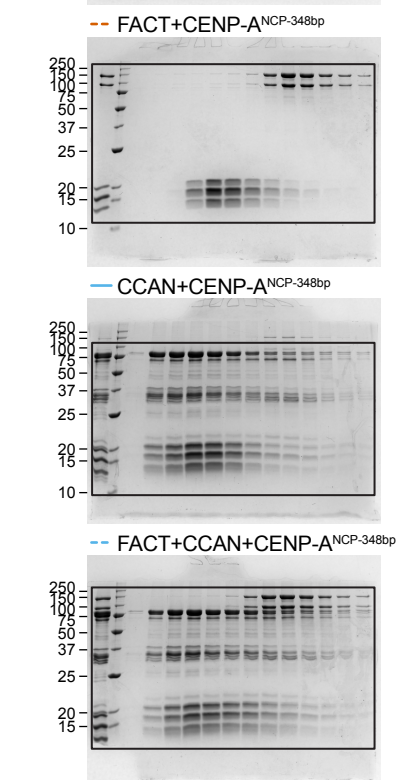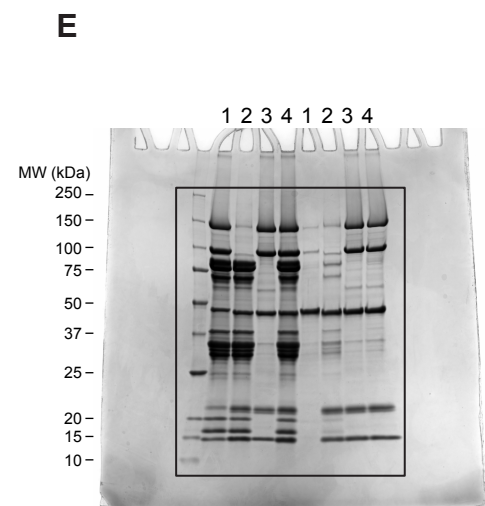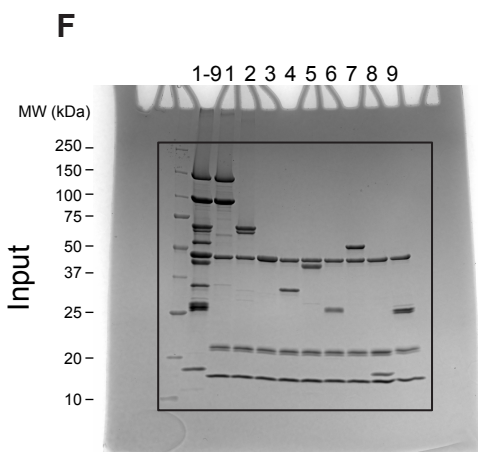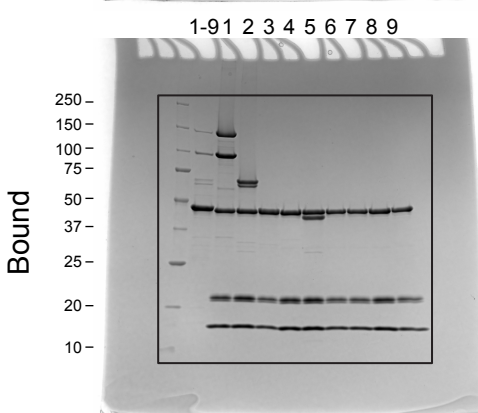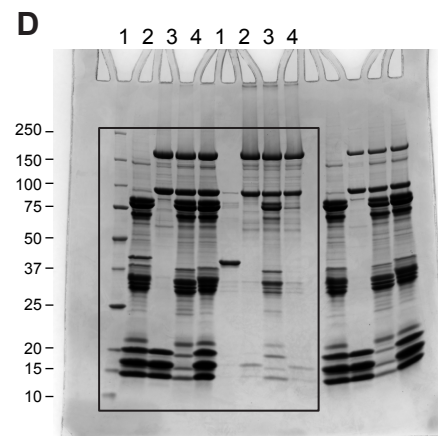

Supplement: SourceData FS6 — is the source file for Fig. S6. [file jcb_202412042_sourcedatafs6.pdf]
